# Case report: Analysis of BRCA1 and BRCA2 gene mutations in a hereditary ovarian cancer family

**DOI:** 10.1007/s10815-020-01783-w

**Published:** 2020-04-30

**Authors:** Ying Liao, Chunhua Tu, Xiaoxia Song, Liping Cai

**Affiliations:** 1 Department of Gynecology, Xinyu People’s Hospital, Xinyu, 338000 Jiangxi China; 2 Department of Obstetrics and Gynecology, The First Affiliated Hospital of Nanchang University, No. 17 Yongwai Zhengjie, Nanchang, 330000 Jiangxi China

**Keywords:** Chinese hereditary ovarian cancer family, Breast cancer susceptibility gene 1/2 mutations, BRCA 1/2 gene screening

## Abstract

**Objective:**

Breast cancer susceptibility gene 1/2 (BRCA1/2) is the most important susceptibility gene associated with hereditary ovarian cancer (HOC). We aimed to screen BRAC1 and BRAC2 gene mutations in a member of a hereditary ovarian cancer family in China, and to analyze the structure and function of the mutant protein.

**Methods:**

A typical HOC family was selected. Blood samples and pathological tissue samples were taken from the female members of the family. Blood samples from two patients with sporadic ovaries of the same pathological type were taken as a control group. After RNA extraction, PCR amplification was applied and the PCR products were directly sequenced and aligned, prediction and analysis of protein structure and molecular conformation that may be caused by BRCA1/2 mutation.

**Results:**

The whole gene analysis of BRCA1 and BRCA2 in ovarian cancer patients in the family showed that there were 8 mutations in BRCA1 whole gene sequencing, including 3 nonsense mutations (2314C>T, 2543T>C, 4540T>C); two mutations have been recorded, which are associated with cervical cancer (2844C>T) and endometriosis (3345A>G); three newly discovered mutations (3780A>G, 5069A>G, 3326A>T). Among them, 3780A>G and 5069A>G caused amino acid changes, while 3326A>T mutation caused Arg mutation to stop codon. A total of 7 mutations were detected in BRCA2 whole-genome sequencing, including 5 non-significant mutations (3623A>G, 4034T>C, 4790A>G, 6740G>C, 7469A>G); one no-record mutation (1716T>A), and 1 recorded mutation (1342A>C), which was associated with breast cancer and ovarian cancer. BRCA1 (3326A>T) and BRCA2 (1342A>C) mutations were co-existing in patients (II1, II3, and II5) identified as serous adenocarcinoma grade II. Two cases of ovarian serous cystadenocarcinoma with no history of family tumors were normalized for BRCA1/2 gene sequencing. In the gene detection of III generation female, four females with BRCA2 (1342A>C) mutation were found, and one of them also carried the BRCA1 (3326A>T) mutation, who can be considered a high-risk group of HOC in this family. Online protein structure predictions revealed that BRCA1 (3326A>T) mutations mutated AGA at this site to TGA resulting in a translated Arg (arginine) mutation as a stop codon, while BRCA2 (1342A>C) mutated AAT at this site to CAT resulting in a translated Asn mutation to His.

**Conclusion:**

The BRCA1 (3326A>T) and BRCA2 (1342A>C) were detected in the HOC family, which may be the susceptibility gene of the family’s HOC. The BRCA1/2 gene screening may be possible to obtain high-risk populations in this family.

**Electronic supplementary material:**

The online version of this article (10.1007/s10815-020-01783-w) contains supplementary material, which is available to authorized users.

## Introduction

Ovarian cancer is one of the three major malignant tumors in gynecology, and its mortality rate ranks first in gynecological tumors [1]. Due to the lack of characteristic early symptoms and effective screening methods, late detection and poor prognosis are recognized features of ovarian cancer. Recent studies have found that genetic factors are important risk factors for ovarian cancer, and 10 to 15% of ovarian cancers belong to hereditary ovarian cancer syndrome (HOCS) [2]. HOCS conforms to autosomal dominant inheritance that often aggregates in the family. The age of onset of HOCS is relatively early that it can be combined with tumors such as breast cancer, colorectal cancer, and endometrial cancer to form a family-wide tumor pathogenesis model [3].

HOCS is associated with mutations in germ cell-associated susceptibility genes, and the penetrance rate is high. Detection of related gene mutations has become an effective means of screening for high-risk HOCS populations [4]. Current research confirms that breast cancer susceptibility gene 1/2 (BRCA1/2) is the most important susceptibility gene associated with HOCS [5, 6]. BRCA1 and BRCA2 gene mutations account for 10~18% of ovarian cancer [7]. The cumulative risk of ovarian cancer in the BRCA1 and BRCA2 mutation carriers was 40% and 18%, respectively, at age 70, which was much higher than 1.4% in the general population [8]. Mutations in the BRCA1/2 gene also increase the risk of pancreatic cancer, gastric cancer, and prostate cancer [9]. Detection of BRCA1/2 gene mutations contributes to screening and early diagnosis of HOCS patients [10]. Detection of BRCA gene mutations in high-risk populations to identify progenitor mutations or high-frequency mutation sites has important guiding significance for the assessment of the risk of BRCA1/2 mutation carriers.

In the USA and Europe, the detection of BRCA1/2 mutation has become an important screening tool for high-risk populations of ovarian cancer [11, 12]. However, the study of BRCA1/2 gene mutations in patients with high-risk HOC is not deep in China. Although hundreds of mutation sites have been found in BRCA1/2, not all mutations are associated with ovarian cancer. There are no reports on the common mutation sites of the BRCA1/2 gene in Chinese HOC. In the present study, BRAC1 and BRAC2 mutations were screened for members of a typical HOC family, and structural and functional analysis of the mutant protein was performed. Our study is a useful exploration of the oncogenic detection of HOC in Chinese population.

## Materials and methods

### Research object

A typical HOC family was selected from the First Affiliated Hospital of Nanchang University. This family is consistent with the definition of HOCS by Lynch et al. [13]. A comprehensive questionnaire survey was conducted among all women in the family and the relationship among relatives was repeatedly checked. The proband (II5), female, born in 1972, was admitted to the First Affiliated Hospital of Nanchang University on March 8, 2012. During the hospitalization, II5 underwent TP regimen with neoadjuvant chemotherapy and ovarian cancer reduction. Pathologically confirmed as stage IIIc of ovarian serous cystadenocarcinoma, II5 died in October 2014 due to systemic metastasis of ovarian cancer. II1 and II3 were similar to II5 and were diagnosed as bilateral ovarian serous adenocarcinoma grade III. II1 underwent tumor cytoreductive surgery in August 2012, and B-ultrasound, chest radiographs, and tumor markers were performed every 3 months after surgery for regular follow-up. II3 underwent ovarian cancer cytoreductive surgery in April 2007 and died in March 2011. II4 was diagnosed with rectal cancer in 2000 and died in 2001 (not included in the study due to incomplete clinical data). The clinical data of the proband and other ovarian cancer patients are complete. According to the family survey questionnaire, the female body index of the family is normal, no menarche is too early or too late, and the lactation period ranges from 8 to 12 months. There is no ovulation or hormone replacement therapy. All members of the family have no bad eating habits and exposure to radioactive materials. There was no history of endometriosis, breast, and colorectal disease in the affected women (II5) before the onset of illness. Histopathological examination was confirmed by two deputy chief physicians and above experts in the Department of Pathology, the First Affiliated Hospital of Nanchang University. Blood samples were taken from the female members of the family, and pathological tissue samples were taken from the deceased patients. Blood samples from 2 patients with sporadic ovaries of the same pathological type were taken as a control group. There was no family history of cancer in the control group of 3 generations of relatives. The study was approved by the Ethics Committee of the First Affiliated Hospital of Nanchang University, and all subjects signed informed consent. Authors had access to information that could identify individual participants during or after data collection. The patient’s pathology information can be found in the supplementary file 1.

### DNA extraction

For the surviving subjects, peripheral blood DNA was extracted using whole blood DNA extraction kit (QIAGEN, China) and stored at − 20 °C. For the deceased subjects, DNA of paraffin-embedded tissues obtained from previous operations was extracted with paraffin-embedded tissue DNA extraction kit (QIAGEN, China) and stored at − 20 °C. All operations are carried out in strict accordance with the manufacturer’s instructions.

### Primer design

The exon primers of BRCA1 and BRCA2 were designed using primer3 online primer design software based on the sequence information of each point on NCBI. The primer sequences were in the supplementary file 2.

### Polymerase chain reaction

The coding regions, the exon and intron splicing regions, and the 5′ and 3′ non-coding regions of BRCA1 and BRCA2 were subjected to PCR amplification, respectively. The PCR test kit was supplied by Takara (Japan). PCR amplification conditions: 95 °C(3 min), 95 °C (30 s), 60 °C (30 s), 72 °C (30 s), 72 °C (50 s), a total of 10 cycles; 95 °C (30 s), 58 °C (40 s), 72 °C (50 s), a total of 12 cycles; 95 °C (30 s), 58 °C (40 s), 72 °C (50 s), a total of 10 cycles; 72 °C extension 10 min, 4 °C preservation. Five microliters of the PCR product was electrophoresed on a 1.0% agarose gel at a constant pressure of 120 V for 30 min. The imager was used to observe, record, and save the results.

### Analysis

The sequencing results were directly compared with BLAST using the PUBMED website. Sequencing peak maps were aligned using DNASTAR_Lasergene.v7.1 software. The mRNA base sequence is translated into an amino acid sequence using DNASTAR software. Online protein structure prediction and analysis were using SWISS-MODEL and PUBMED.

## Results

### Pedigree of the HOC family

**Fig. 1:**
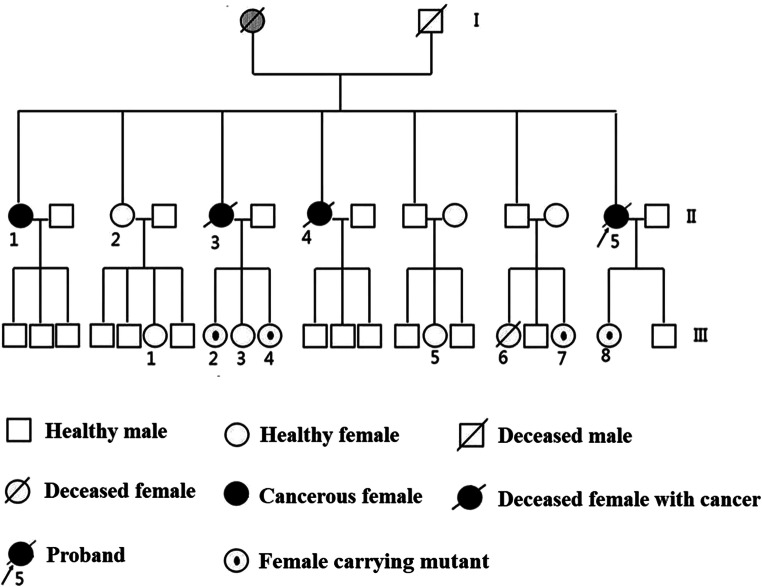
Pedigree of hereditary ovarian cancer family

There were 3 cases of ovarian cancer (II1, II3, and II5) and 1 case of rectal cancer (II4) in the II generation of women in this family, and II2 was healthy. The remaining family members have no abnormal symptoms. The proband (II5) in the family was diagnosed as bilateral ovarian serous adenocarcinoma grade III at the age of 40, and died after surgery and conventional chemoradiotherapy, and the survival period was 31 months. II1 and II3 were similar to the proband and were diagnosed as bilateral ovarian serous adenocarcinoma grade III. II3 was diagnosed with ovarian cancer at the age of 50 and had a survival of 48 months. II4 was diagnosed with rectal cancer and was not included in the study because of imperfect clinical data. The pedigree location of 3 patients with ovarian cancer in the family was shown in Fig. 1.

### Mutation screening of II generation in the HOC family

We first performed a full-gene analysis of BRCA1 and BRCA2 in II1, II2, and II5. A total of 8 mutations were found in the whole gene sequencing of BRCA1, including 3 nonsense mutations (2314C>T, 2543T>C, 4540T>C); two mutations have been recorded, which are associated with cervical cancer (2844C>T) [14] and endometriosis (3345A>G) [15], respectively; three new mutations (3780A>G, 5069A>G, 3326A>T) were found that no literature has been reported. Among the three newly discovered mutations, 3780A>G and 5069A>G caused amino acid changes, while the 3326A>T mutation caused Arg mutation to a stop codon. The mutation coexisting in II1 and II5 was 3326A>T.

A total of 7 mutations were detected in the whole gene sequencing of BRCA2, among which 5 were non-significant mutations (3623A>G, 4034T>C, 4790A>G, 6740G>C, 7469A>G), one mutation (1716T>A) not recorded, and one recorded mutation (1342A>C) associated with breast cancer [16] and ovarian cancer [17]. The mutation coexisting in two ovarian cancer patients, II1 and II5, was 1342A>C. Additionally, BRCA1 (3326A>T) and BRCA2 (1342A>C) mutations were also detected in tumor tissues of II3.

Taken together, the 3326A>T heterozygous mutation of BRCA1 gene and the 1342A>C heterozygous mutation of BRCA2 gene were detected in the confirmed II generation ovarian cancer patients, but none of those in healthy generation II was found.

### Mutation screening of III generation in the HOC family

According to the results of the II generation, we compared the gene sequencing of 3326 bases of BRCA1 gene and the 1342 bases of BRCA2 gene in all females of the III generation in the family and 2 patients with sporadic serous ovarian cancer (control). No cases of ovarian cancer had been found in the III generation female in this family, but genetic tests have shown that there were 1342A>C single mutations in the BRCA2 gene in III2, III4, and III7, and there were two mutations of BRCA1 (3326A>T) and BRCA2 (1342A>C) in III8. However, no genetic mutations were found in the genetic tests of the control group.

### Analysis of protein multiple sequences

According to the amino acid sequence of BRCA1 and BRCA2 proteins in the PUBMED website, we selected 9 species including *Arabidopsis thaliana*, fish, rodents, dogs, cattle, monkeys, orangutans, and baboons for homology analysis of BRCA1 and BRCA2 protein sequences. We found that the BRCA1 and BRCA2 genes are highly conserved across species.

### Prediction and analysis of online protein structure

The heterozygous mutation of BRCA1 (3326A>T) mutated the codon AGA at this position to TGA, resulting in a translated Arg mutation as a stop codon, which ultimately resulted in termination of translation, protein shortening, and molecular conformational changes.

The heterozygous mutation of the BRCA2 (1342A>C) mutated the codon AAT at this position to CAT, resulting in the translation of the translated Asn (polar neutral amino acid) to His (positively charged basic amino acid), which ultimately caused a change in the stability of the molecular model.

## Discussion

With the research progress of tumor susceptibility gene screening, the selection of genetic counseling and gene mutations in high-risk populations through family history has become a routine way to screen HOCS [18]. This study collected a typical HOC family and performed an internationally recognized sequencing analysis of the BRCA1/2 gene associated with HOC in all women in the family. We found that three women with ovarian cancer (II1, II3, and II5) in the family carried BRCA1 (3326A>T) and BRCA2 (1342A>C) mutations, and no BRCA1 and BRCA2 mutations were detected in healthy individuals. Therefore, the family can be diagnosed as a family of HOC, and the detected BRCA1 (3326A>T) and BRCA2 (1342A>C) mutations may be oncogenes. The daughter of the proband (III8) carries both BRCA1 (3326A>T) and BRCA2 (1342A>C) mutations, and three females (III2, III4, and III7) carried the BRCA2 (1342A>C) mutation, which can be considered a high-risk group of HOC in this family. We recommend that patients with carcinogenic genes in this family who are not already ill should be closely monitored.

The BRCA1 gene is located on chromosome 17q21 and encodes a protein of 1863 amino acids to participate in the process of gene regulation and subsequent DNA repair [19]. The BRCA2 gene is located at 13q12 and encodes a protein of 3418 amino acids responsible for localization of cloning and translation and subsequent transcription [20]. BRCA1/2 is a tumor suppressor gene and plays an important role in regulating the replication of human cells, DNA damage repair of genetic material, and normal cell growth, whose mutation can result in changes in the corresponding biological functions. The BRCA1/2 gene mutation sites vary in different regions and ethnic groups, and the ancestral mutations and mutation rates are also different, which is caused by genetic background differences [21]. There are hundreds of mutations in BRCA1/2 that have been found. About 50% of hereditary breast cancers and 70~80% of hereditary ovarian cancer patients have BRCA1 mutations in their germ cells, while sporadic ovarian cancers have a BRCA1 mutation rate of less than 5% [22]. The risk of breast cancer and ovarian cancer in BRCA2 mutation carriers is 50~85% and 10~20%, respectively [23]. It has been reported that most of the BRCA1/2 gene mutations associated with HOCS are concentrated in the 10th and 11th exon regions [24]. At present, the data on the mutation distribution of these two genes in high-risk groups are mostly from European and American populations, and the research on Chinese population is less or not comprehensive. In this study, we performed a sequencing analysis of the BRCA1/2 gene in the second-generation ovarian cancer patients in the selected HOCS family.

BRCA1 can undergo multiple forms of multi-site mutations that its mutations are scattered throughout the coding sequence [25]. BRCA1 has been detected with more than 300 different mutations and polymorphisms, of which 88% are small fragment insertions and deletions (box shifts) or nonsense mutations, resulting in the occurrence of a truncated protein in advance of the stop codon. The other 12% were splicing site variants, regulatory factor mutations, missense mutations, or polymorphisms. Most of the mutations at the amino or carboxy terminus of the protein result in the destruction of zinc finger structures or BRCT repeats, and these two structures play an important role in the tumor suppressor function of BRCA1. The most common mutations in the BRCA1 gene include S1613G on exon 16 and P871L on exon 11 in combination with E1038G, which were found in breast cancer families in India, Greece, Turkey, and Italy. Studies in the Norwegian population have shown that 1675delA and 1135insA account for one-third of hereditary breast cancer ovarian cancer [26], while 816delGT and 3347delAG account for 68% of Norwegian BRCA1 mutation carriers [27]. S1631G is mutated from Ser to Gly due to amino acid exchange, and this mutation site was found in the BRCA1 mutation carrier in Italy [28]. Some scholars have reported that the 1100detAT and 5589del mutations of the BRCA1 gene are more common in the Chinese population [29]. Shen [30] found that BRCA1: 5589del8 has a high incidence rate in the Chinese population, which may be a high-frequency mutation point with partial ancestor effect. However, our results show that the mutation hotspots are different from those in other regions, which may be related to the complex genetic background of Chinese population. We found 8 mutations in the BRCA1 gene of the selected HOCS family of ovarian cancer patients (6 located in exon 11). In addition to 3 nonsense mutations, there were 2 mutations associated with cervical cancer (2844C>T) [14] and endometriosis (3345A>G) [15], respectively. Moreover, we also found three new unrecorded mutations, 3780A>G, 5069A>G, and 3326A>T. Interestingly, the mutation that coexisted in patients with ovarian cancer (II1, II3, and II5) was 3326A>T. This missense mutation is located in exon 10 and no report on this mutation has been reported so far. We performed a comparison of various species of amino acids near this mutation point, and found that this amino acid sequence is highly conserved. BRCA1 (3326A>T) mutation mutated Arg to a stop codon, leading to early termination of protein chain translation and protein truncation, which also directly led to the deletion of many important structural functional regions such as BRCT domain, and the molecular model conformation has undergone tremendous changes. These regions play important roles in regulating cell cycle, DNA double-strand damage repair, and cell proliferation and apoptosis [31]. We speculated that the BRCA1 (3326A>T) mutation may be a susceptibility gene for ovarian cancer in this family.

The most common mutation in BRCA2 is protein truncation caused by frame shift, which ultimately leads to loss of protein activity. The S1832P, T2766I, N2781I, and K2860T mutations in BRCA2 were found to be associated with breast cancer in the Danish population [32]. There was also a 9023A>C mutation that causes the BRCA2 protein structure to be replaced by Pro to His. Additionally, nonsense mutations found in Chilean breast cancer families may interfere with normal BRCA2 function [33]. In the Romanian breast cancer, the 4817A>G mutation in exon 11 of the BRCA2 gene was found to change the Lys residue to Arg, which is considered to be a pathogenic mutation [34]. BRCA2 S2834X and 5802del4 are commonly reported in the Japanese population [35]. A multicenter study in China showed that 3109C>T of BRCA2 is a possible ancestral mutation [36]. We found 7 mutations in the BRCA2 gene (3 of which are located in exon 10 and 4 in exon 11), of which 1342A>G has been shown to be associated with breast cancer [37] and ovarian cancer [17]. Most importantly, we found that the 1342A>C mutation coexists in II1 and II5, and this missense mutation is located on exon 11. We hypothesized that BRCA2 (1342A>C) mutations are associated with the development of ovarian cancer in this family. We found that the BRCA2 (1342A>C) mutation makes Asn mutate to His at this position. The positive charge carried by His interferes with the hydrogen bonding of the region, and the benzene ring structure contained in His destroys the structure of the region, which ultimately may also lead to the stability of the protein. Palli et al. reported that specific BRCA2 N372H (1342A>C) mutations increase the risk of breast cancer in women [16]. Su et al. found that the BRCA2 N372H polymorphism is associated with susceptibility to ovarian cancer, especially serous subtypes of ovarian cancer [17]. We hypothesized that BRCA2 (1342A>C) mutation may be one of the potential causes of HOC in this family.

Although BRCA1 and BRCA2 have no homology in the amino acid sequence, they have certain commonality in inhibiting tumors. These two genes have a special structural region RAD51 [38]. RAD51 is one of the members of the protein family that mediates the exchange of DNA strands leading to normal recombination that is considered to be a decisive functional junction that links recombination, repair, and cell cycle checkpoint. RAD51 not only specifically interacts with the BRC sequence encoded by BRCA2 exon 11 but also presents in the S phase of cells together with BRCA1 [39]. We found in this family that BRCA1 (3326A>T) caused the mutation of 1031 Arg to a stop codon, which resulted in the deletion of the C-terminal domain “BRCT” of the 1650~1724 and 1763~1842 amino acid residues. There are many DNA damage repair and cell cycle checkpoint proteins in the BRCT domain, and mutations make it impossible to locate accurately. Moreover, this region is also the binding site of RAD51 and BRCA2, which causes the binding of BRCA1 to RAD51 and BRCA2 to be blocked. Furthermore, the 1342A>C mutation of BRCA2 gene leads to abnormal structure of BRCA2 protein, which cannot bind to RAD51, RAD52, P53, etc., so that the regulatory points of cell cycle cannot be accurately located, and the damaged DNA double strand cannot be repaired, which may lead to tumor occurrence [40]. Therefore, we speculated that the mutations between BRCA1 and BRCA2 synergistically lead to the occurrence of ovarian cancer in this family. However, mutations in the BRCA1/2 gene are “autosomal dominant inheritance,” meaning that not all mutation carriers develop cancer, but only those with this mutation have high cancer susceptibility [41]. We have both mutations coexisting in the III generation of women in the family, as well as detecting the presence of a single mutation. We believed that screening and follow-up of BRCA1/2 gene mutations in III generation women and their offspring may have important implications for the occurrence of ovarian cancer in this family members. In addition, the occurrence of breast cancer may be the result of the combined action of multiple susceptible genes, such as TP53, ATM, PTEN, STK11, CDH1, BRIP1, PALB2, RAD50, HER2, and C-myc [42]. Kimbung et al. confirmed that BRCA1 mutant breast cancer is usually accompanied by a mutation in the PTEN gene [43]. Moreover, BRCA1 gene mutations are also related to heterozygosity and microsatellite instability RAD52, RAD54, and RAD54B genes [44]. Therefore, in subsequent studies, we need to select more mutation genes related to breast cancer in order to further improve the detection and monitoring of susceptible genes in high-risk populations in this HOC family.

However, there are still many shortcomings in this study. First, our sample size is limited to one family of hereditary ovarian cancer. Secondly, only the detection of BRCA1/2 gene mutation was selected in the ovarian cancer family to speculate on the type of gene mutation that may be caused by the family. We should also improve the genome-wide detection and observe whether other related genes have abnormal mutations in the future. Additionally, we will collect more HOC families in subsequent studies to enrich the data of the prominent characteristics of BRCA gene in Chinese population.

In summary, in this study, all female members of a typical HOC family were studied, and BRCA1 (3326A>T) and BRCA2 (1342A>C) mutations were detected, which may be the causative genes of ovarian cancer in this family. We speculated that the synergy of these two mutations may together lead to the occurrence of ovarian cancer in this family. Genetic screening of women in this family for suspected oncogenes can obtain high-risk populations of ovarian cancer in this family, which is important for the diagnosis and treatment of high-risk women in the family.

## Electronic supplementary material

ESM 1 (DOCX 1822 kb)

ESM 2 (DOCX 23 kb)
