# Supplementary material for: Case report: Analysis of BRCA1 and BRCA2 gene mutations in a hereditary ovarian cancer family
Source: J Assist Reprod Genet. 2020 Apr 30;37(6):1489–95. doi: 10.1007/s10815-020-01783-w (PMC7311593; doi:10.1007/s10815-020-01783-w)
Supplement: Supplementary file 1 — (DOCX 1822 kb) [file 10815_2020_1783_MOESM1_ESM.docx]

Case Report: analysis of BRCA1 and BRCA2 gene mutations in a hereditary ovarian cancer family

Pathological analysis

After oophorectomy, the pathological tissues of the proband (II5) were collected for analysis (Figure 1). As a result, cancerous metastases were detected in the bilateral fallopian tubes, the two uterines, the posterior peritoneum, and the appendix serosa. The invasion of the cancerous tissue from the serosa and the serosa from the bottom of the uterus and the muscular layer can be seen. There was no cancer involvement in the stump of the vagina and the omentum. There were 10 right obturator lymph nodes, 2 of which showed cancer metastasis (2/10); Four para-aortic lymph nodes, 1 metastasis (1/4).The endometrium was in a secretory state, the cervix was chronically inflamed, and squamous hyperplasia was accompanied by retention cysts.

Figure 1. Pathological tissue analysis of the proband (II5).

(A) Ovarian tissues (×100). (B) Uterine metastases (×100). (C) Lymph node metastasis (×100). (D) Fallopian tube metastases (×100).

After oophorectomy, the pathological tissues of II3 were collected for analysis (Figure 2). It was found that no metastasis was found in the parauterine tissue and right fallopian tube on both sides, while cancer involvement was found in the left fallopian tube, appendix serosa, and omentum. There were 11 inguinal inguinal lymph nodes in the left condyle, and 5 showed metastasis (5/11).


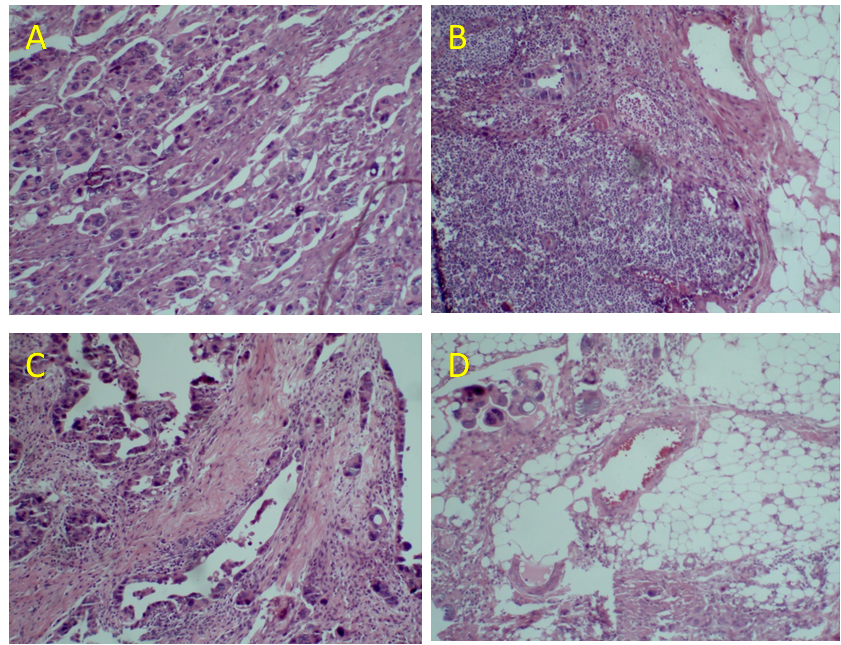


Figure 2. Analysis of pathological tissue specimens of II3.

(A) Ovarian tissues(×100). (B) Lymph node metastasis(×100). (C) Fallopian tube metastases(×100). (D) Omental metastases(×100).
