# Supplementary material for: Case report: Analysis of BRCA1 and BRCA2 gene mutations in a hereditary ovarian cancer family
Source: J Assist Reprod Genet. 2020 Apr 30;37(6):1489–95. doi: 10.1007/s10815-020-01783-w (PMC7311593; doi:10.1007/s10815-020-01783-w)
Supplement: Supplementary file 2 — (DOCX 23 kb) [file 10815_2020_1783_MOESM2_ESM.docx]

**Case Report: analysis of BRCA1 and BRCA2 gene mutations in a hereditary ovarian cancer family**

The exon primers of BRCA1 and BRCA2 were designed using primer3 online primer design software based on the sequence information of each point on NCBI. The primer sequences are as follow (Suppplementary Tables 1-4).

Suppplementary Table 1. The 5’ primer sequences for BRCA1.

| Gene | Exon no. | 5' Primer name | Sequence |
| --- | --- | --- | --- |
| BRCA1 | Exon 1 | BRCA1_E1_F | ttctgagaggctgctgctta |
|  | Exon 2 | BRCA1_E2_F | agctaaggctaccaccacct |
|  | Exon 3 | BRCA1_E3_F2 | tgaaagctcactgaaggtaagg |
|  | Exon 4 | BRCA1_E4_F | tttgtggtgaggtgttgaga |
|  | Exon 5 | BRCA1_E5_F  BRCA1_E6_F | gctgtggatttagggcagta  cccactggtctcacacctta |
|  | Exon 6 |  |  |
|  | Exon 7 | BRCA1_E7_F | gccccagacattttagtgtg |
|  | Exon 8 | BRCA1_E8_F | ccatgcctttaaccacttct |
|  | Exon 9 | BRCA1_E9_F | tgaactcatgacctcaagtgg |
|  | Exon 10 | BRCA1_E10_F | aggtgtggtttctgcatagg |
|  |  | BRCA1_E10_F2 | aaagcaaacagcctggctta |
|  | Exon 11  Exon 11  Exon 12 | BRCA1_E10_F3 | ggaactaaccaaacggagca |
|  |  | BRCA1_E10_F4 | cccaagggactaattcatgg |
|  |  | BRCA1_E10_F5 | cagagggccaaaattgaatg |
|  |  | BRCA1_E10_F6 | ggcactcaggaaagtatctcg |
|  |  | BRCA1_E10_F7 | ccaccactttttcccatcaa |
|  |  | BRCA1_E11_F | cacacagctaggacgtcatct |
|  |  | BRCA1_E12_F | ctatttgggggaatttgagg |
|  | Exon 13 | BRCA1_E13_F | gtctgttgcattgcttgtgt |
|  | Exon 14 | BRCA1_E14_F | agatatggtgcctcagacca |
|  | Exon 15 | BRCA1_E15_F | ctttggatttccaccaacac |
|  | Exon 16 | BRCA1_E16_F | tacctccagcctgggtgac |
|  | Exon 17 | BRCA1_E17-18_F | ttctgtcaccaggggtttta |
|  | Exon 18 | BRCA2_E17_F | accatgctcagcaatgaagt |
|  | Exon 19 | BRCA1_E19_F | gaggtttcaccatgttggtc |
|  | Exon 20 | BRCA1_E20_F | atgcctgtagagtgcaggtc |
|  | Exon 21 | BRCA1_E21_F | gaaggaaggtgggtgttttt |
|  | Exon 22 | BRCA1_E22_F2 | ggaggctgagatggaaggat |
|  | Exon 23 | BRCA1_E23_F | gcaagactccgtctcaaaaa |

Suppplementary Table 2. The 3’ primer sequences for BRCA1.

| Gene | Exon no. | 3' Primer name | Sequence |
| --- | --- | --- | --- |
| BRCA1 | Exon 1 | BRCA1_E1_R | ccctcgcgacctacaaact |
|  | Exon 2 | BRCA1_E2_R | gagtggatggagaacaagga |
|  | Exon 3 | BRCA1_E3_R2 | ccaggaactatgattacaaccaa |
|  | Exon 4 | BRCA1_E4_R | aggcagatgtcccataaaac |
|  | Exon 5 | BRCA1_E5_R  BRCA1_E6_R | gtgtgagaccagtgggagtaa  ccacagagcaagactccatc |
|  | Exon 6 |  |  |
|  | Exon 7 | BRCA1_E7_R | gtgggaactgcgtcttttac |
|  | Exon 8 | BRCA1_E8_R | gggaagagggagaaattcag |
|  | Exon 9 | BRCA1_E9_R | acgaaagggcaacaatcagt |
|  | Exon 10 | BRCA1_E10_R | ggagtccgcctatcattaca |
|  |  | BRCA1_E10_R2 | aggttcagctttcgttttgaa |
|  | Exon 11  Exon 11  Exon 12  Exon 11  Exon 12 | BRCA1_E10_R3 | cttgtttcccgactgtggtt |
|  |  | BRCA1_E10_R4 | ctcaggttgcaaaaccccta |
|  |  | BRCA1_E10_R5 | tgctccccaaaagcataaac |
|  |  | BRCA1_E10_R6 | ctgaccaaccacaggaaagc |
|  |  | BRCA1_E10_R7 | gaacaaacctgagatgcatga |
|  |  | BRCA1_E11_R | tgagttccatcaaggtgctt |
|  |  | BRCA1_E12_R | agttgtgagcagggacaaga |
|  | Exon 13 | BRCA1_E13_R | ccgcacatttctcatgttgt |
|  | Exon 14 | BRCA1_E14_R | accaagactccctcatcctc |
|  | Exon 15 | BRCA1_E15_R | agatgccttctggggaataa |
|  | Exon 16 | BRCA1_E16_R | cgaccacacccagctaattt |
|  | Exon 17 | BRCA1_E17-18_R | aagtggtgcattgatggaag |
|  | Exon 18 | BRCA2_E17_F | accatgctcagcaatgaagt |
|  | Exon 19 | BRCA1_E19_R | aaagtcccagctcttccact |
|  | Exon 20 | BRCA1_E20_R | catgttggcactaacagcag |
|  | Exon 21 | BRCA1_E21_R | ccttacccatcccttacaga |
|  | Exon 22 | BRCA1_E22_R2 | tcaaaaagacattttagccattca |
|  | Exon 23 | BRCA1_E23_R | caagggagacttcaagcaga |

Suppplementary Table 3. The 5’ primer sequences for BRCA2.

| Gene | Exon no. | 5' Primer name | Sequence |
| --- | --- | --- | --- |
| BRCA2 | Exon 1 | BRCA2_E1_F | cagatactgacggttgggatg |
|  | Exon 2 | BRCA2_E2_F | ctgttaccgttccaggagatg |
|  | Exon 3 | BRCA2_E3_F | cacctttctatagattcgcaagaga |
|  | Exon 4 | BRCA2_E4_F | cctcttcttacaactcccta |
|  | Exon 5 | BRCA2_E5-6_F | ccagcagctgaaatttgtgagt |
|  | Exon 6 |  |  |
|  | Exon 7 | BRCA2_E7_F | acctttgccctgagatttacaa |
|  | Exon 8 | BRCA2_E8_F | tgcctttaggctgtagtgttg |
|  | Exon 9 | BRCA2_E9_F | ggtgcaagatttcatcacac |
|  | Exon 10 | BRCA2_E10_F1 | aatcaggggaatcaggcttt |
|  |  | BRCA2_E10_F2 | tgccacgtatttctagcctacc |
|  | Exon 11 | BRCA2_E11_F1 | actgtgcccaaacactaccttt |
|  |  | BRCA2_E11_F2 | ctgagcaagcctcagtcaatta |
|  |  | BRCA2_E11_F3 | gctgttgccacctgaaaaat |
|  |  | BRCA2_E11_F4 | cttctgcagaggtacatccaa |
|  |  | BRCA2_E11_F5 | tgtggtgccacctaagctct |
|  |  | BRCA2_E11_F6 | ggccacctgcatttaggata |
|  |  | BRCA2_E11_F7 | cagcaagtggaaagcaagttt |
|  | Exon 12 | BRCA2_E12_F | ctgtggta-tctggtagcatctg |
|  | Exon 13 | BRCA2_E13_F | tgatcatctggctgtgtcag |
|  | Exon 14 | BRCA2_E14_F | atggcaaccatggtgaatac |
|  | Exon 15 | BRCA2_E15_F | agggtttctccattttggtc |
|  | Exon 16 | BRCA2_E16_F | ggtgg-aaaaggtacagcaga |
|  | Exon 17 | BRCA2_E17_F | accatgctcagcaatgaagt |
|  | Exon 18 | BRCA2_E18_F | ccactatttggggattgcta |
|  | Exon 19 | BRCA2_E19_F4 | tcgaagttccttttatctgttttct |
|  | Exon 20 | BRCA2_E20_F | atgttggtcaggctgatctc |
|  | Exon 21 | BRCA2_E21_F | ctcccttctttgggtgtttt |
|  | Exon 22 | BRCA2_E22_F | gtttgaggcacctgagaata |
|  | Exon 23 | BRCA2_E23-24_F | tccactactaatgcccacaa |
|  | Exon 24 |  |  |
|  | Exon 25 | BRCA2_E25_F | gcactgtaagcaacaggtca |
|  | Exon 26 | BRCA2_E26_F | actttttctctgttcccctctc |
|  | Exon 27 | BRCA2_E27_F | gggagggagactgtgtgtaata |

Suppplementary Table 4. The 3’ primer sequences for BRCA2.

| Gene | Exon no. | 3' Primer name | Sequence |
| --- | --- | --- | --- |
| BRCA2 | Exon 1 | BRCA2_E1_R | gagggaagttaagtcttgagctg |
|  | Exon 2 | BRCA2_E2_R | ggttaacctgcaaacgatgatt |
|  | Exon 3 | BRCA2_E3_R | agaggccagagagactgatttg |
|  | Exon 4 | BRCA2_E4_R | aaccagccaattcaacat |
|  | Exon 5 | BRCA2_E5-6_R | aattgcctgtatgaggcagaa |
|  | Exon 6 |  |  |
|  | Exon 7 | BRCA2_E7_R | aatgcttgacaccactggacta |
|  | Exon 8 | BRCA2_E8_R | taccccatgaataggggact |
|  | Exon 9 | BRCA2_E9_R | tgcatgcttgtaatctcagc |
|  | Exon 10 | BRCA2_E10_R1 | agaagccactggagaagttcc |
|  |  | BRCA2_E10_R2 | tgtaacaaacctgcacatcctg |
|  | Exon 11 | BRCA2_E11_R1 | caaattgcttgctgctgtctac |
|  |  | BRCA2_E11_R2 | caatgactgaataagggactg |
|  |  | BRCA2_E11_R3 | gacctaagagtcctgcccatt |
|  |  | BRCA2_E11_R4 | tcccgctagctgtatgaaaa |
|  |  | BRCA2_E11_R5 | tcgtttggcaaatttttgatt |
|  |  | BRCA2_E11_R6 | tgggtttctcttatcaacacga |
|  |  | BRCA2_E11_R7 | tcaaaccatactcccccaaa |
|  | Exon 12 | BRCA2_E12_R | tgtcaactttcggaagatcc |
|  | Exon 13 | BRCA2_E13_R | actgattcggagcaatttcc |
|  | Exon 14 | BRCA2_E14_R | cagcagtcctagattgtgtgtc |
|  | Exon 15 | BRCA2_E15_R | cattcatccattcctgcact |
|  | Exon 16 | BRCA2_E16_R | ttaaaccccaggacaaacag |
|  | Exon 17 | BRCA2_E17_R | gtgggatggcaactgtcac |
|  | Exon 18 | BRCA2_E18_R | ctctggacctcccaaaaact |
|  | Exon 19 | BRCA2_E19_R4 | gctgcagtgaaccaagatca |
|  | Exon 20 | BRCA2_E20_R | gacctgatatttctgtcccttg |
|  | Exon 21 | BRCA2_E21_R | gaaaacataccaccacactcg |
|  | Exon 22 | BRCA2_E22_R | gggcattagtagtggattttgc |
|  | Exon 23 | BRCA2_E23-24_R | caaatttgccaactggtagc |
|  | Exon 24 |  |  |
|  | Exon 25 | BRCA2_E25_R | ctgaggttcatgggcaatta |
|  | Exon 26 | BRCA2_E26_R | gtgcacccagagtttcatatct |
|  | Exon 27 | BRCA2_E27_R | cgatacacaaacgctgaggta |
